# Introducing and integrating perinatal mental health screening: Development of an equity‐informed evidence‐based approach

**DOI:** 10.1111/hex.13526

**Published:** 2022-05-24

**Authors:** Rebecca Blackmore, Jacqueline A. Boyle, Kylie M. Gray, Suzanne Willey, Nicole Highet, Melanie Gibson‐Helm

**Affiliations:** ^1^ Monash Centre for Health, Research and Implementation School of Public Health and Preventive Medicine, Monash University Melbourne Australia; ^2^ Centre for Educational Development, Appraisal and Research (CEDAR), Faculty of Social Sciences University of Warwick Coventry UK; ^3^ Department of Psychiatry, School of Clinical Sciences, Centre for Developmental Psychiatry and Psychology Monash University Melbourne Australia; ^4^ Centre of Perinatal Excellence (COPE) Melbourne Australia; ^5^ Centre for Women's Health Research, Te Tātai Hauora O Hine Faculty of Health, Victoria University of Wellington Wellington New Zealand

**Keywords:** early pregnancy, health services research, maternity services, perinatal, psychiatry, psychology

## Abstract

**Background:**

Pregnancy is a time of increased risk for developing or re‐experiencing mental illness. Perinatal mental health screening for all women is recommended in many national guidelines, but a number of systems‐level and individual barriers often hinder policy implementation. These barriers result in missed opportunities for detection and early intervention and are likely to be experienced disproportionately by women from culturally and linguistically diverse backgrounds, including women of refugee backgrounds. The objectives of this study were to develop a theory‐informed, evidence‐based guide for introducing and integrating perinatal mental health screening across health settings and to synthesize the learnings from an implementation initiative and multisectoral partnership between the Centre of Perinatal Excellence (COPE), and a university‐based research centre. COPE is a nongovernmental organization (NGO) commissioned to update the Australian perinatal mental health guidelines, train health professionals and implement digital screening.

**Methods:**

In this case study, barriers to implementation were prospectively identified and strategies to overcome them were developed. A pilot perinatal screening programme for depression and anxiety with a strong health equity focus was implemented and evaluated at a large public maternity service delivering care to a culturally diverse population of women in metropolitan Melbourne, Australia, including women of refugee background. Strategies that were identified preimplementation and postevaluation were mapped to theoretical frameworks. An implementation guide was developed to support future policy, planning and decision‐making by healthcare organizations.

**Results:**

Using a behavioural change framework (Capability, Opportunity, Motivation–Behaviour Model), the key barriers, processes and outcomes are described for a real‐world example designed to maximize accessibility, feasibility and acceptability. A Programme Logic Model was developed to demonstrate the relationships of the inputs, which included stakeholder consultation, resource development and a digital screening platform, with the outcomes of the programme. A seven‐stage implementation guide is presented for use in a range of healthcare settings.

**Conclusions:**

These findings describe an equity‐informed, evidence‐based approach that can be used by healthcare organizations to address common systems and individual‐level barriers to implement perinatal depression and anxiety screening guidelines.

**Patient or Public Contribution:**

These results present strategies that were informed by prior research involving patients and staff from a large public antenatal clinic in Melbourne, Australia. This involved interviews with health professionals from the clinic such as midwives, obstetricians, perinatal mental health and refugee health experts and interpreters. Interviews were also conducted with women of refugee background who were attending the clinic for antenatal care. A steering committee was formed to facilitate the implementation of the perinatal mental health screening programme comprising staff from key hospital departments, GP liaison, refugee health and well‐being, the NGO COPE and academic experts in psychology, midwifery, obstetrics and public health. This committee met fortnightly for 2 years to devise strategies to address the barriers, implement and evaluate the programme. A community advisory group was also formed that involved women from eight different countries, some of refugee background, who had recently given birth at the health service. This committee met bimonthly and was instrumental in planning the implementation and evaluation such as recruitment strategies, resources and facilitating an understanding of the cultural complexity of the women participating in the study.

## 1 BACKGROUND

Perinatal mental health has been acknowledged by the World Health Organization as a significant public health issue directly impacting maternal morbidity, obstetric outcomes and infant attachment and development.^1^, ^2^, ^3^, ^4^ Pregnancy is a critical time with respect to mental health as there is an increased chance of women experiencing or re‐experiencing mental illness.^5^, ^6^, ^7^ Associated long‐term costs as a result of perinatal mental illness, including costs to healthcare, well‐being, productivity and intergenerational impact, have been assessed at $5.2 billion in Australia^8^ and £8.1 billion pounds per year in the United Kingdom.^9^

During the perinatal period, defined as conception to 12 months following birth, depression and anxiety are the most commonly experienced mental illnesses.^10^, ^11^ For women living in high‐income countries, perinatal depression and anxiety are reported to affect up to 15.0%.^11^, ^12^ For women living in low‐ and middle‐income countries, the prevalence of perinatal depression is more than double this rate, with a pooled prevalence of 31% for any depressive illness.^13^ In particular, women of refugee background are at an even greater risk of mental illness during pregnancy.^14^, ^15^ This is attributable to the conflict, trauma, separation from family and protracted situations of uncertainty that are hallmarks of refugee experiences.^15^

Screening for depression and anxiety during pregnancy is justified to improve health outcomes for women and their children. Participation in perinatal depression screening programmes has been shown to improve identification of women at risk, referral uptake and engagement with services, which in turn has a positive impact on mental health outcomes.^16^ The Edinburgh Postnatal Depression Scale (EPDS) has been validated to detect symptoms of both perinatal depression and anxiety and is the recommended screening measure in the Australian clinical guidelines.^5^ Routine, standardized screening in pregnancy for mental illness is recommended in high‐income countries including the United Kingdom,^17^ the United States of America^18^ and Australia.^5^ However, this is a common evidence‐practice gap, with screening poorly implemented due to a lack of knowledge and support for women and health professionals.^19^ Additionally, there are a number of systems‐level and individual barriers including short consultation times, inadequate funding, absence of clear referral pathways and insufficient mental health training.^20^ This represents a critical gap in best‐practice pregnancy care, resulting in the under‐recognition of women at risk of perinatal mental illness.

To ensure that screening is successful and improves healthcare, the barriers to implementation must be addressed for all women, including those with complex care needs or who commonly experience additional barriers to accessing best‐practice care, such as women of refugee background. Additional challenges to perinatal mental health screening when working with refugee populations can include availability of face‐to‐face interpreters, health literacy and cultural barriers such as the stigma of disclosing symptoms of mental illness.^20^

Implementing sustainable change within healthcare settings presents a number of complex challenges. The aim of this paper is to provide a summary of the strategies devised and delivered to address the complex nature of implementation. A perinatal depression and anxiety screening programme using a digital screening platform implemented at a large public maternity service in metropolitan Melbourne is presented as the exemplar, with additional relevance to services with multicultural populations, or populations with the potential for complex risk profiles. Barriers and enablers to implementation were identified during formative research and stakeholder consultation preceding initial design of the programme.^20^ Further strategies were developed iteratively during implementation to address additional barriers as they arose. An evaluation of the programme yielded further refinements for large‐scale, sustainable roll‐out. Here, we bring the learnings from each stage and interpret them together for the first time. This novel synthesis uses the Theoretical Domains Framework (TDF),^21^ a Programme Logic Model^22^ and the Capability, Opportunity, Motivation–Behaviour Model (COM‐B)^23^ to further understand the barriers and required interventions for successful behaviour change. Concurrent with the implementation and evaluation of this programme, a perinatal mental health screening implementation guide was developed to further assist health services implement perinatal mental health guidelines in their specific context.

## 2 METHODS

The Standards for Reporting Implementation Studies (StaRI)^24^ checklist was used to report this study.

### 2.1 Setting

We used a case study approach^25^ to describe how the perinatal depression and anxiety screening programme was designed and implemented. The protocol for the evaluation of this programme has been published elsewhere,^26^ but in summary, the study was conducted at a public antenatal clinic located in a large teaching hospital within south‐eastern Melbourne, Australia. The area is home to a large multicultural and refugee population. This clinic was designed to specifically cater for women of refugee background 1 day/week, supported by a Refugee Health Nurse Liaison (RHNL) and two bicultural workers. Approximately half of the women who attended this clinic were of refugee background or considered to be refugee‐like, that is, arrived in Australia on a spousal visa from a refugee‐source country including Afghanistan, Myanmar, the Republic of South Sudan and Sri Lanka. This study was approved by the Monash Health Human Research Ethics Committee (14475L).

### 2.2 Implementation strategy development

#### 2.2.1 Data collection

The development of the implementation strategies was informed by the findings of prior research with stakeholders. This involved semi‐structured interviews with health professionals (*n* = 28) such as midwives, obstetricians, perinatal mental health and refugee health experts, interpreters as well as women of refugee background (*n* = 9).^20^ The results from this study identified the barriers and enablers within eight key TDF domain constructs.^21^

#### 2.2.2 Stakeholder engagement

A steering committee was then formed to facilitate implementation. The committee comprised of staff from key hospital departments, GP liaison, refugee health and well‐being, the nongovernmental organization (NGO) COPE and academic experts in psychology, midwifery, obstetrics and public health. This committee met fortnightly for 2 years to devise strategies to address the barriers, implement and evaluate the programme. The committee addressed concerns of the research team or hospital staff as they arose and responded with practical solutions. In addition, a community advisory group was also formed and comprised of women from eight different countries, some of refugee backgrounds, with most having recently had a baby at the health service. This committee met bimonthly and was instrumental in planning the implementation and evaluation such as recruitment strategies, resources and facilitating an understanding of the cultural complexity of the women participating in the study.

#### 2.2.3 Evaluation

**Figure 1:**
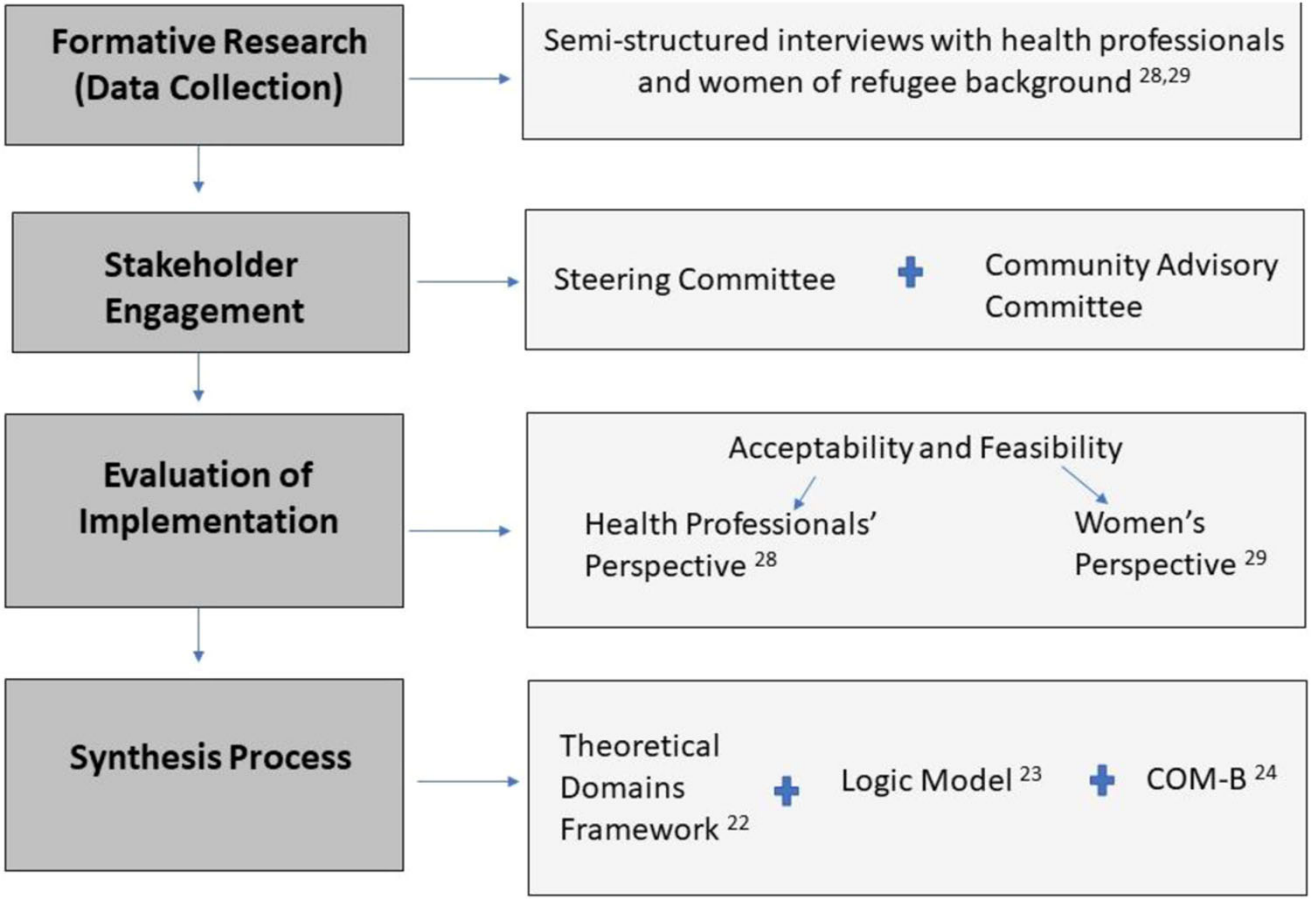
Flow chart describing the process of the stages of implementation and evaluation.

The acceptability and feasibility of the perinatal depression and anxiety screening programme in the antenatal period were evaluated from the both the perspective of the health professionals as well as women of refugee background, and these results have been published elsewhere.^27^, ^28^ In summary, an explanatory and sequential mixed‐methods approach incorporating surveys, interviews and focus groups enabled a holistic view and enriched understanding of the perceptions and experiences of health professionals and women with respect to perinatal mental health screening.^27^, ^28^ Health professionals completed an online survey (*n* = 38), two focus groups: one with nine participants and the other with four (*n* = 13), and eight semi‐structured interviews with a total of 11 participants), five of which were completed individually due to their unique roles within the clinic. The evaluation of health professionals was guided by the Normalization Process Theory.^29^ The perspectives of women of refugee background were evaluated using focus groups (*n* = 1; five participants) and semi‐structured telephone interviews (*n* = 17). The findings of the evaluation led to the development of strategies required for programme refinement and scale‐up. In the synthesis presented here, these strategies are described and interpreted using implementation theories and behaviour change frameworks for the first time (Figure 1).

### 2.3 Synthesis process

Here, we bring the learnings from each stage and interpret them together for the first time. This novel synthesis uses three frameworks, the TDF,^21^ a Programme Logic Model^22^ and the COM‐B,^23^ to further understand the barriers and required interventions for successful behaviour change. These frameworks provide a synthesis of research and learnings to better understand the relationships between barriers and strategies, inputs and outcomes associated with implementing perinatal depression and anxiety screening.

**Figure 2:**
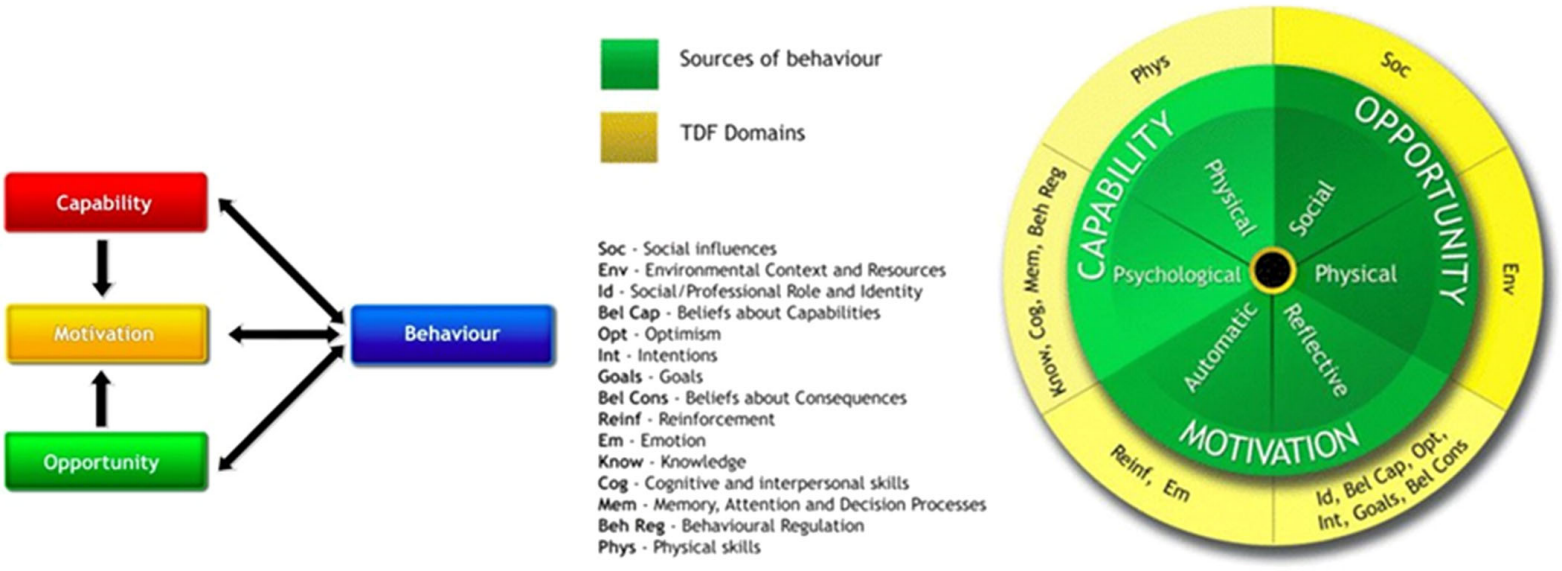
Map of Theoretical Domains Framework (TDF) to the COM‐B model. Figure reproduced from Alexander et al.^31^ COM‐B, Capability, Opportunity, Motivation–Behaviour Model.

1. TDF
  The TDF is an integrated framework of theoretical constructs that are related to behaviour change.^21^ The TDF was used to map the strategies and barriers devised before, during and after implementation.
2. Programme Logic Model
  A programme logic model was developed to demonstrate the relationships of the inputs, outputs and outcomes of the perinatal mental health screening programme.^22^ Logic models are useful in evaluating how outcomes are produced by processes (inputs); however, a logic model alone is not sufficient enough to capture the dynamics of complex interventions.^30^ Therefore, the theoretical framework of the COM‐B model was applied to understand the requirements for behaviour change.
3. The COM‐B
  The COM‐B model is part of the Behaviour Change Wheel.^23^ It proposes that for someone to engage in a behaviour, they must be physically and psychologically able (capability), have the social and physical opportunity to do the behaviour (opportunity) and want to do the behaviour more than other competing behaviours (automatic and reflective motivation).^23^ As each of the TDF domains relates to a component of the COM‐B model (Figure 2), when incorporated together, this provides greater understanding of behaviour change to improve implementation.

### 2.4 Developing an implementation guide: Translation of evidence into practice

The Centre of Perinatal Excellence (COPE) was represented on the steering committee from the screening programme's inception. COPE is a not‐for‐profit organization in perinatal mental health and the NGO commissioned to update the Australian national perinatal mental health guidelines and implement innovative digital screening across healthcare settings. Based on the learnings from this pilot screening programme, as well as implementation of the digital platform across other healthcare settings, COPE has developed a guide to assist healthcare services in implementing perinatal mental health screening.

## 3 RESULTS

Using a case study approach, we present the barriers to implementing perinatal mental health screening as identified by the formative research conducted during the design and implementation phases.^20^, ^27^, ^28^ The strategies devised to address the said barriers are also presented, using the TDF domains: knowledge and skills, social/professional role and identity, beliefs about capabilities and consequences, environmental context and resources, social influences and behavioural regulation.

### 3.1 Identified barriers and strategies for implementation

#### 3.1.1 Knowledge and skills

##### Preimplementation barriers

Health professionals acknowledged the importance of routine antenatal mental health screening. However, a lack of knowledge and skills regarding mental health screening measures, administration and scoring and the specific health needs of women of refugee background was identified as a barrier to screening.^20^

##### Preimplementation strategies

Training for all staff in the antenatal clinic, including midwives, obstetricians and administrative staff, was delivered as part of existing regular staff meetings within the organization. In attendance, members of the research team, a midwife and two psychologists, provided training on perinatal depression and anxiety, suicide risk assessment, administration and scoring of the recommended screening measure, the EPDS,^32^ use of the digital platform iCOPE^33^ and codesigned referral pathways. Aspects of this training addressed the health needs of women of refugee background in addition to improving staff understanding of refugee experiences. Midwives were also provided access to the online Basic Skills in Perinatal Mental Health course delivered by COPE, which provides the knowledge and skills required to undertake perinatal mental health screening and use of the iCOPE digital screening platform.

##### Strategies developed during implementation

Staff training was delivered before implementation as well as ongoing access to onsite research staff with relevant knowledge regarding perinatal mental healthcare, EPDS administration and use of the digital platform iCOPE for one‐on‐one consultancies. Existing weekly staff meetings were used to discuss process issues as they arose, facilitating a collaborative approach and shared decision‐making responsibilities for managing issues, challenges and noting successes.

#### 3.1.2 Social/professional role and identity

##### Preimplementation barriers

Health professionals identified the need for a ‘go‐to’ person within the healthcare service to support both staff and women in accessing referrals to improve referral uptake and service usage.^20^

##### Preimplementation strategies

Collaborating with the organization's refugee health service by including the manager as a member of the project steering committee enabled the mobilization of key personnel. A RHNL supported staff in the clinic and a bicultural worker telephoned women before their appointment to introduce the research project as well as asking them to attend 15 min earlier to complete screening. Bicultural workers were employed by the health service as a point of contact for women to assist with navigation within a potentially complex and unfamiliar health system.^27^ They enabled cross‐cultural understanding of the women attending the clinic, many of whom shared similar cultural backgrounds.

#### 3.1.3 Beliefs about capabilities

##### Preimplementation barriers

The challenges of incorporating the additional task of mental health screening into routine midwifery practice were identified as a barrier. There were concerns that existing time pressures for antenatal appointments would be further exhausted by the addition of manual administration and scoring of the EPDS. There is acknowledgement that these time pressures could contribute to errors when manually calculating EPDS scores. The error rate of manual scoring for the EPDS is reported to be up to 28.9%.^34^ Health professionals recommended the need for a streamlined and efficient screening and referral process as well as access to a support person onsite.^20^

##### Preimplementation strategies

The digital screening platform, iCOPE, developed by the COPE was specifically designed to streamline and improve the efficiency of mental health screening.^33^ It allowed women to complete the screening measure on their own before the antenatal appointment and on a tablet. It takes approximately 6–10 min to complete, slightly longer if an interpreter is used. On completion, the iCOPE programme automatically calculated and provided healthcare professionals with a clinical report including the overall EPDS score, response to Item 10 (self‐harm and suicidality) and the anxiety subscale score (based on responses to Items 3, 4 and 5) and individual response items, eliminating scorer error. The report also included a clinical management guide to assist with discussion of results and initiate referral based on codesigned referral pathways developed for the programme. The iCOPE programme also generated score‐based, language appropriate information for women postscreening with links to further information in response to the answers provided.

##### Strategies developed during implementation

During the implementation of the programme, ‘organizational champions’ were recognized, described as staff identified by the research team as early adopters of the screening programme and quick to adapt to the implemented changes. These ‘organizational champions’ fulfilled the role of an onsite support person and further facilitated a supportive practice environment. They were able to engage midwives who were experiencing difficulties with aspects of the implementation or showing signs of reluctance to engage.

#### 3.1.4 Beliefs about consequences

##### Preimplementation barriers

The stigma surrounding the disclosure of mental illness symptoms and interpreter confidentiality were both described as potential barriers to screening and accessing services.^20^ Health professionals advocated for the normalization of mental health screening and the need for a sensitive approach.

##### Preimplementation strategies

During the codesign process of the screening programme, stakeholders raised the importance of describing the screening programme as a key component of routine pregnancy care, sensitively presenting the options of mental health services, and providing follow‐up care that was perceived as useful and appropriate. The training that was delivered to all staff in the antenatal clinic included aspects of how best to introduce mental health screening, the use of suitable and nonthreatening terminology and reiterating that screening was part of standard and routine care for all women. Referral options included a refugee health and well‐being service that was both multidisciplinary and culturally competent and expected to overcome some of the barriers to referral uptake.

##### Strategies developed during implementation

Further targeted training was deemed necessary to support midwives in administering Item 10 on the EPDS, which assesses the risk of self‐harm and/or suicidality. Further training was arranged with the provision flowcharts provided for clear referral pathways to manage positive responses to Item 10.

#### 3.1.5 Environmental context and resources

##### Preimplementation barriers

It was unanimously agreed by health professionals that in addition to the provision of professional and trustworthy female interpreters, phone interpreters were inappropriate for mental health screening.^20^ Health professionals identified the importance of translated screening measures, but raised concerns over the quality of translations available for the EPDS.^20^ Literal translation of the EPDS can be problematic and the best practice process of translation involves forward and back translation and testing.^35^

##### Preimplementation strategies

After identifying the key languages of the women attending the clinic, existing EPDS translations that had been validated were obtained and new translations were conducted using best‐practice methods.^35^ For one identified language, Dari, the investigation of the reliability and validity of this version formed an additional part of the programme. The clinic provided face‐to‐face interviews with female interpreters, experienced in maternity care and perinatal mental health screening.

##### Barriers arising during implementation

Women with low literacy were not able to complete self‐administered screening. The Burmese EPDS version was unable to load onto the digital screening platform due to issues with the script.

##### Strategies planned for scale‐up

The provision of translation audio versions of the EPDS will allow women with low literacy, and those whose preferred language is Burmese, to have the option of self‐administration.^27^, ^28^

#### 3.1.6 Social influences

##### Preimplementation strategies

Women of refugee background often lack the social supports that most women and families rely on during pregnancy and after the arrival of a baby. Therefore, continuity of care was identified as critical for building rapport and trust to facilitate honest disclosure between the women and their healthcare professionals.^20^

##### Barriers arising during implementation

Due to staffing constraints experienced in most public antenatal clinics, continuity of care can be challenging. Regular staff turnover in the clinic meant that there was an ongoing need to ensure that new staff received training and support to include screening as part of their routine practice.

##### Strategies developed during implementation

The presence of a ‘go‐to’ person such as the RHNL acted to assist staff with screening and women with referrals and follow‐up. The RHNL fulfils the role of regular clinic contact for staff and women when there is a need to discuss mental health services and referrals. The ‘organizational champions’ were critical in maintaining enthusiasm for the programme and engaging new staff.

#### 3.1.7 Behavioural regulation

##### Preimplementation barriers

The health professionals identified the need for immediate follow‐up for positive responses to the screening questions on self‐harm and suicidality.^20^ Clear referral guidelines were requested in clinic rooms to ensure appropriate referrals.^20^

##### Strategies developed during implementation

Referral pathways were codesigned by the steering committee with options for referrals dependent on EPDS scores, as well as for women of refugee or nonrefugee backgrounds. This included training for the health professionals in the clinic on risk management and assessing suicidality. Clear referral pathways also allowed for improved ease of both short‐ and long‐term follow‐up regarding referrals as well as improved communication between services. Stakeholder consultation identified relevant and appropriate services within the community to be included in the referral pathways. Clear and explicit flowchart diagrams were developed to ensure a clear process for referrals including appropriate management of self‐harm and suicidality risk.

### 3.2 Programme Logic Model

**Figure 3:**
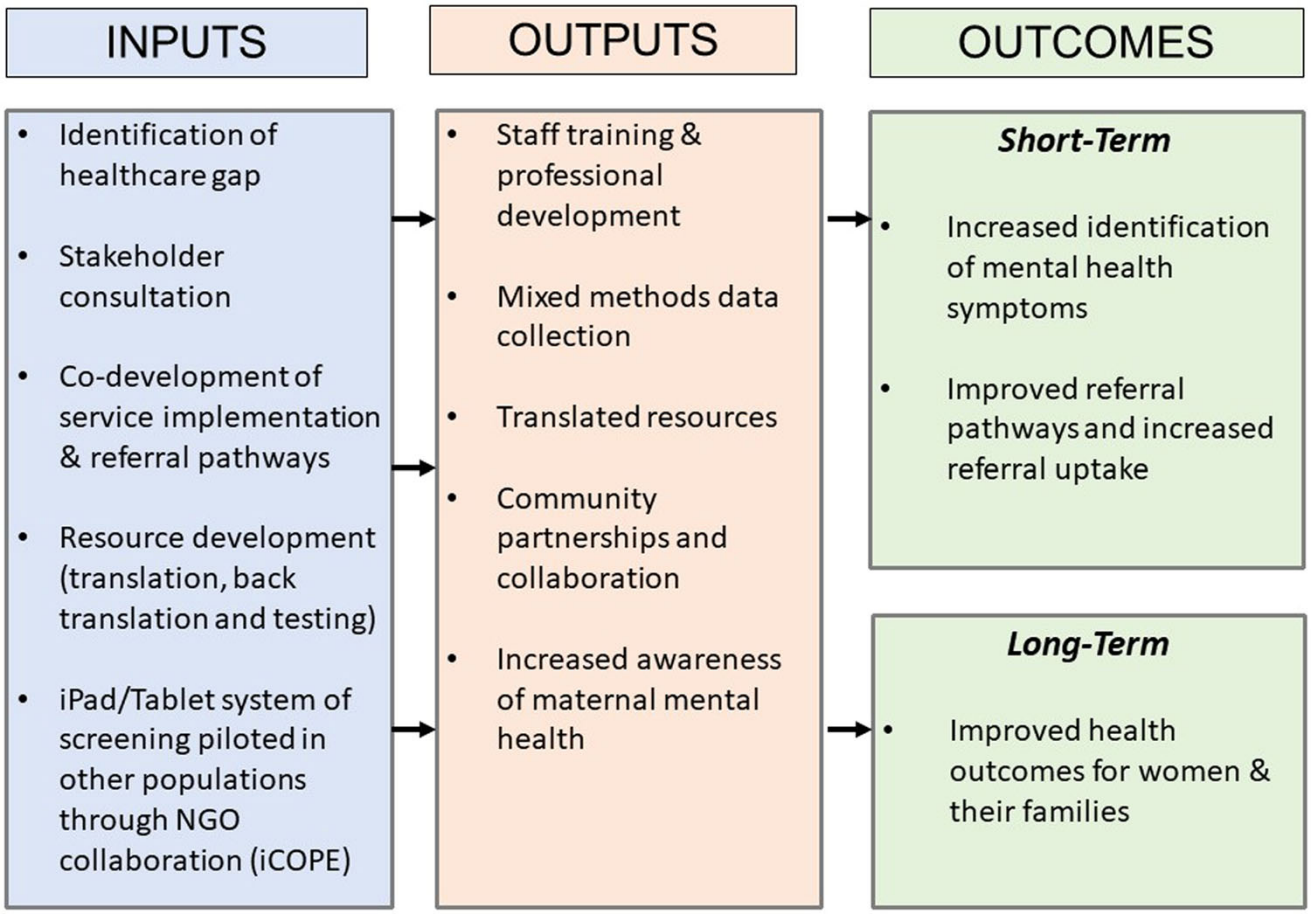
Programme Logic Model of the perinatal mental health screening programme.

The logic model designed for the programme (Figure 3) describes all of the featured elements (inputs and outputs) that were involved in implementing the perinatal mental health screening programme along with the proposed short‐ and long‐term outcomes.

### 3.3 The COM‐B

Using the behavioural change framework of the COM‐B model, the key barriers, processes and outcomes are described.^23^ The application of this framework highlights the importance of a multistrategy approach to behaviour change that delivers strategies that address the multiple factors at play (Table 1).

**Table 1:** Applying the COM‐B model^23^ to the creation of a perinatal mental health screening programme.

Capability	Motivation	Opportunity
An individual's physical (skills, strength, stamina) and psychological (knowledge, psychological skills) capacity to engage in the behaviour.	Processes that affect ability to do the behaviour at the required time Motivation is reflective (self‐conscious planning) and automatic (processes related to wants and needs, desires).	Factors that affect the behaviour in the context of the environment: physically (time, triggers, resources, physical barriers) and socially (interpersonal influences, social cues, cultural norms).
**Psychological capability**	**Reflective motivation**	**Physical opportunity**
**Knowledge**: Knowledge of how to implement best‐practice antenatal care (clinical guidelines) Knowledge and experience of mental health screening measures (e.g., staff training) **Behavioural regulation**: Follow‐up (short and long term) of women as a result of screening practices Clear referral guidelines (e.g., referral flowcharts available in clinic rooms)	**Beliefs about consequences**: Small group of trained and experienced interpreters (e.g., ensuring confidentiality and consistency) Sensitive approach to screening to reduce stigma surrounding disclosure of mental health symptoms Screening introduced as part of normal routine antenatal care and for all women **Beliefs about capabilities**: Efficient screening process (e.g., iCope platform) Clear referral process (e.g., staff training) Onsite support staff (e.g., identification of organizational ‘champions’)	**Environmental context & resources**: Availability of face‐to‐face interpreters Interpreters experienced and trained in mental health and EPDS High‐quality translated screening measures Time‐efficient screening (e.g., self‐administration) Privacy for screening (e.g., self‐administration)
**Physical capability**	**Automatic motivation**	**Social opportunity**
**Skills**: Training in the ‘How to’ of screening, recognition of symptoms, referral pathways Understanding of refugee experiences and specific health needs	**Social/professional role and identity**: ‘Go‐to’ person for staff and supporting women to access referrals Bicultural workers to improve cultural appropriateness of screening and follow‐up	**Social influences**: Identifying potential social supports for women of refugee background Continuity of care in the clinic to facilitate honest discussions and referral uptake (e.g., ‘go‐to’ person)

Abbreviations: COM‐B, Capability, Opportunity, Motivation–Behaviour Model; EPDS, Edinburgh Postnatal Depression Scale.

### 3.4 Implementation guide

**Figure 4:**
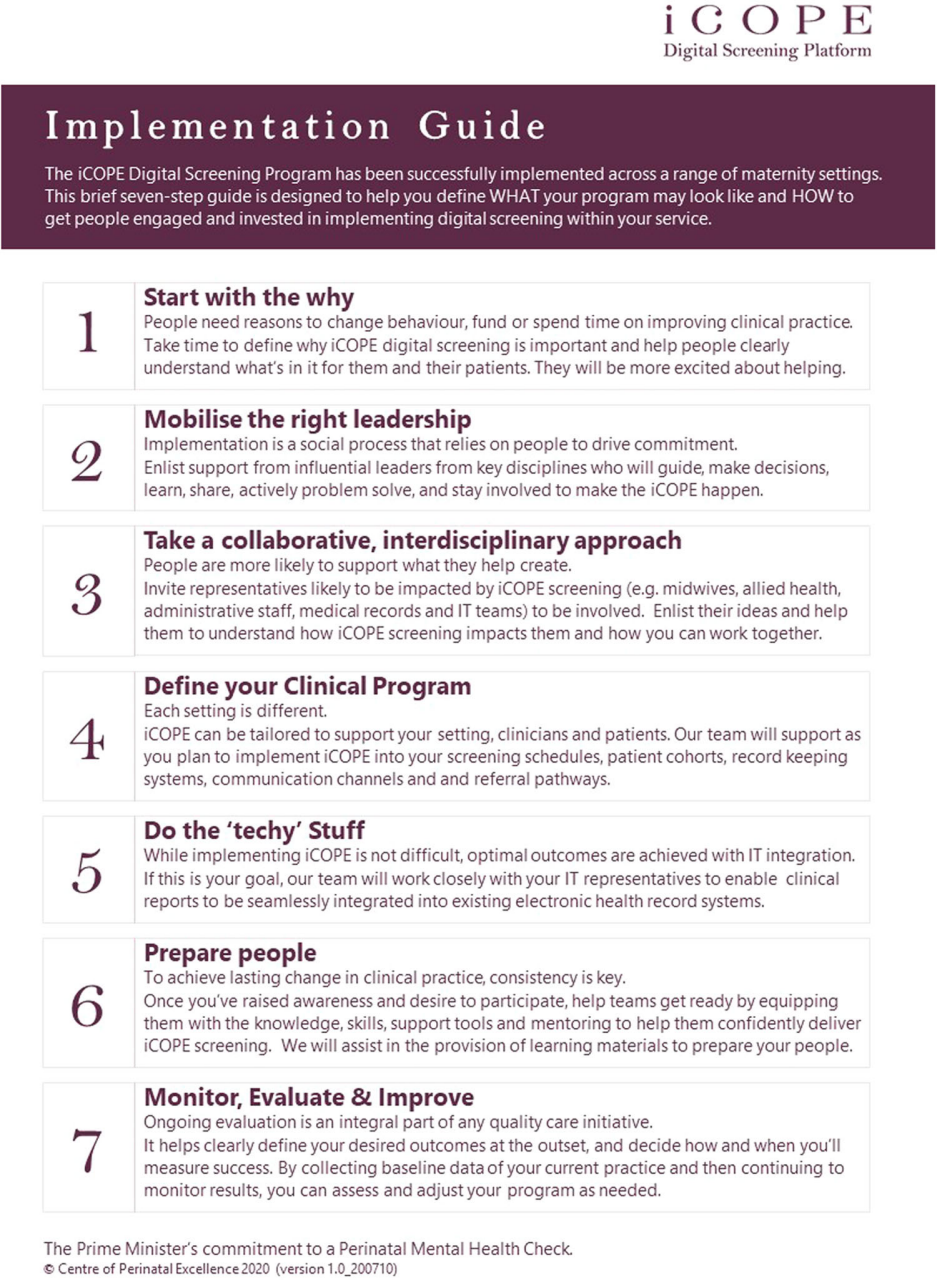
COPE implementation guide for perinatal mental health screening. COPE, Centre of Perinatal Excellence.

The iCOPE digital platform was designed to facilitate depression and anxiety screening in healthcare settings during pregnancy by ensuring consistent and accurate interpretation of clinical scales in accordance with international clinical guidelines.^33^ COPE was involved in all stages of the pilot screening programme from design through to delivery, including involvement in the steering committee and evaluation of the programme. Based on the experiences of this pilot programme as well as implementation of their digital screening platform across other healthcare settings, they have developed an implementation guide to assist healthcare services in implementing perinatal mental health screening (Figure 4).

## 4 DISCUSSION

A case study approach was used to describe how a perinatal depression and anxiety screening programme using a digital screening platform was designed and implemented at a large public maternity service in metropolitan Melbourne. This study provides additional relevance to health services with multicultural populations, or populations with the potential for complex risk profiles. This paper brings together the formative research and stakeholder consultation that preceded the initial design of the programme and presents the barriers and enablers to implementation.^20^ This novel synthesis brings together the learnings from each stage of project design and development, resulting in a summary of strategies that were devised and delivered to address the complex nature of implementing perinatal mental health screening programmes. For the first time, the barriers to implementation and resulting strategies have been described in the context of three frameworks, the TDF, a Programme Logic Model and the COM‐B, further enhancing understanding of the barriers and required interventions for successful behaviour change.

To identify women at risk of perinatal depression and anxiety, there is a need to develop perinatal mental health screening programmes, grounded in behaviour change theory, that can be delivered across a range of antenatal care settings and with populations with complex needs. To improve health equity in perinatal care, the barriers to implementation must be addressed for all women, including those who experience additional barriers such as women of refugee background. Perinatal mental health screening programmes that are designed to promote equitable access are not only critical to improving health outcomes for all women but also improving trust between the health service and the women seeking pregnancy care.^36^ This case study provided a valuable opportunity to study how a healthcare organization, in partnership with research and an NGO, could implement perinatal depression and anxiety screening that is appropriate for populations with complex needs.

Perinatal depression screening programmes have been found to be beneficial.^16^ These benefits include increases in referral rates and identification, increases in mental health service usage as well as extending to improvements in parenting outcomes.^16^ To date, routine perinatal mental health screening has been inadequately implemented, creating a critical gap in maternity healthcare.^37^ The implementation of screening programmes within antenatal services is often inconsistent as it requires action from different departments within the health service and across disciplines of health professionals. These findings highlight the importance of a multi‐strategy approach and how successful implementation involves delivering strategies that address the multiple factors involved. The results from this study aim to reduce barriers to screening based on the requirements for behaviour change: *capability* (e.g., staff training, onsite support, clear referral pathways), *opportunity* (e.g., time efficient screening, self‐administration, quality translations of screening measures) and *motivation* (e.g., consistent group of interpreters and sensitive approach to screening).^23^

This case study has a number of strengths. The antenatal clinic was chosen due to its location within one of the largest public health and teaching hospitals that services a large multicultural and refugee population. The learnings from the implementation of the screening programme within this setting may be generalizable to other health service settings, particularly those with diverse populations and complex needs. Ongoing stakeholder involvement facilitated codesign and an ability to evolve as challenges arose. The use of a theory‐informed method enabled a detailed approach to programme design. Despite the many learnings that this programme can provide for other health services, there are some limitations. Screening for perinatal depression and anxiety relies on the availability of healthcare services to provide referral options for women based on their screen scores. A lack of cost‐effective and local mental health service referral options would prevent implementation. It may be challenging to implement a digital screening programme in low‐resource healthcare settings. There were also some issues with loading certain language scripts, such as Burmese, onto the digital platform, resulting in certain languages not being available for women. Audio options of the EPDS would have enabled women with low literacy to complete the screening unassisted and this remains a key focus for future directions. This study remains a case study and further adaption will be required for each unique setting. Although the cost‐effectiveness of implementing a perinatal depression and anxiety screening programme was beyond the scope of this study, it is an area for consideration for future research.

## 5 CONCLUSIONS

Pregnancy is a time of increased likelihood of experiencing mental illness. Women of refugee background are at an even greater risk of mental illness during this period. Therefore, mental health screening during the perinatal period is critically important to achieve the best health outcomes for women and their families. Implementation of perinatal mental health screening has historically been limited due to a number of health service and individual barriers. There is support for screening from both women of refugee background and health professionals, with both groups acknowledging the necessity and importance.^20^, ^27^, ^28^ With the provision of access to digital screening together with this implementation guide, informed by prior research and evaluation, health services within Australia and internationally will be encouraged to use this framework when planning their implementation.

## AUTHOR CONTRIBUTIONS

Melanie Gibson‐Helm, Jacqueline A. Boyle and Kylie M. Gray conceptualized the original study project and design. All authors contributed to the implementation. All authors contributed to material preparation, data collection, analysis and evaluation. Nicole Highet designed and provided the digital screening platform iCOPE. The first draft of the manuscript was written by Rebecca Blackmore, and all authors commented on draft versions of the manuscript. All authors read and approved the final manuscript.

## CONFLICTS OF INTEREST

The authors declare no conflicts of interest.

## Supporting information

Supplementary information.

## Data Availability

Data sharing is not applicable to this article as no new data were created or analysed in this study.
